# Trace elements in *Athyrium distentifolium* from alpine vegetation in the Karkonosze, SW Poland

**DOI:** 10.1007/s10661-020-08438-4

**Published:** 2020-07-03

**Authors:** Aleksandra Kazienko, Karol Torzewski, Bronisław Wojtuń, Aleksandra Samecka-Cymerman, Lucyna Mróz, Alexander J. Kempers

**Affiliations:** 1 Department of Ecology, Biogeochemistry and Environmental Protection, ul. Kanonia 6/8, 50-328 Wrocław, Poland; 2 Department of Environmental Science, Faculty of Sciences, Institute for Water and Wetland, Research Radboud University, Heijendaalseweg 135, 6525 AJ Nijmegen, Netherlands

**Keywords:** Fern, Metal, Altitude, High mountain

## Abstract

The Karkonosze National Park is affected by long-distance metal air transport of anthropogenic contamination as well as by tourist activity. Therefore, concentrations of Cd, Co, Cr, Cu, Fe, Mn, Ni, Pb and Zn were evaluated in soil as well as in vital and non-vital fronds of *Athyrium distentifolium* Opiz collected in the middle of the growing season from glacial cirques in the Karkonosze. Additionally, fronds of the same species turning brown in autumn were collected from the same sampling sites. The health of ferns was impacted by the contents of Co, Cu, Fe, Mn, Ni, Pb and Zn in fronds, which may indicate that *A. distentifolium* uses elements accumulated in its tissues as defence against pathogens. Individuals from higher altitudes contained higher concentrations of Cd, Cu, Ni, Fe and Pb than those from lower altitudes*.* Autumn fronds of *A. distentifolium* contained a significantly higher concentration of Cr, Cu, Fe, Mn and Pb than vital summer fronds, which may indicate sequestration of these elements in senescing fronds probably to remove potentially harmful metals. Non-vital ferns were a better accumulator of Pb than vital ones, while both types of ferns accumulated Mn in a similar way.

**Electronic supplementary material:**

The online version of this article (10.1007/s10661-020-08438-4) contains supplementary material, which is available to authorized users.

## Introduction

The Karkonosze is the largest mountain chain in the Sudety, SW Poland, formed by a crystalline massif reaching up to 1603 m a.s.l. (Urban et al. 2017). Granites, with an admixture of crystalline schists and gneisses, form the main acidic bedrock (Mochnacka and Banaś 2000). Vegetation of the high parts of the Karkonosze is described by Štursa (1998) as arctic-alpine tundra. The longitudinal location of these mountains favours influence of W-E flow (from the former ‘black triangle’) of long-range pollution transport from mining and chemical industries as well as power plants (Pusz 2016; Wojtuń et al. 2013a, [Bibr CR52][Bibr CR53]). One of the constituents of contaminants is nondegradable metals easy accumulated in biota and incorporated in soils with a negative impact on ecosystems (Kabata-Pendias 2001a; Szopka et al. 2011). Accordingly, pollution influence on particular high mountain ecosystems of the Karkonosze must be precisely controlled (Štursa 1998). The best tool which reflects chemistry of the environment is elemental composition in the biomass of plant bioindicators (Holoubek et al. 2000; Schmidt et al. 2010). Ferns are able to survive in polluted soils, especially in active mining areas. They are good indicators, e.g. of arsenic and copper deposits (Kachenko et al. 2007; Chang et al. 2009), or valuable indicators of metalliferous soils (Cornara et al. 2007; Kachenko et al. 2007). According to Kachenko et al. (2007), these plants are able to accumulate also Cd, Cr, Ni, Pb and Zn and may be utilised in the phytoremediation of contaminated soils. Such abilities and additionally tolerance to many environmental extreme factors suggest that ferns may be useful indicators of environmental pollution (Chang et al. 2009). Rybczyński and Mikuła (2011) are also of the opinion that fern gametophytes may be used as models for plant response to various contamination aspects. Studies of the tolerance of terrestrial ferns to metals in the Karkonosze are rather scarce, and therefore, the concentration of trace elements for this research was evaluated in *Athyrium distentifolium* Opiz fronds and soil from its sampling sites*.* The genus *Athyrium* may be an example of ferns with the above-mentioned abilities (Zhang et al. 2014; Kamachi et al. 2015). *A. distentifolium*, the alpine lady-fern, is a circumpolar arctic-montane species growing at high altitude sites. This is a chionophilous fern and thus prefers long-standing snow cover (McHaffie 2005). This author believes that deep snow cover protects *A. distentifolium* from low temperatures in winter and protects early rise in spring. These abilities are an advantage in competition with other species (McHaffie 2005). In the Karkonosze, *A. distentifolium* forms *Athyrietum distentifolii* Hadač 1955 em. W. Mat 1960 association which especially inhabits glacial cirques (Fudali 2010; Żołnierz et al. 2012). *A. distentifolium* is also a dominating species of the understory in the forests of the Karkonosze whose coverage has increased during the industrial era of air pollution (Vacek et al. 1999). However, in the last years, some dieback of the *Athyrietum distentifolii* subalpine tall fern community is observed in the Karkonosze. The cause of the dieback is not known (Dunajski et al. 2016; T. Szymura personal communication). Accordingly, it was examined whether a difference could be observed in the concentration of trace elements in vital and non-vital fronds. The aim of this study was to determine Cd, Co, Cr, Cu, Fe, Mn, Ni, Pb and Zn in soil as well as in both vital and non-vital fronds of *A. distentifolium* collected in the middle of the growing season from glacial cirques and in their vicinity in the Karkonosze. Additionally, fronds of the same plant turning brown in autumn were collected from the same sampling sites. The selected metals were indicated in former investigation as increased contamination in this area due to former ‘black triangle’ long-range emissions (Wojtuń et al. 2013a). Measuring their accumulation in plants contributes to the knowledge of their dispersion and possible protection measures to be taken. The tested hypotheses in this study are the following: (1) Non-vital summer fronds contain significantly higher concentrations of certain trace elements, which may suggest that these metals are accumulated in *A. distentifolium* tissues as defence against pathogens; (2) metal contents in summer green fronds of *A. distentifolium* are significantly lower than those, while they are turning brown in autumn because the excess of metals is withdrawn; and (3) *A. distentifolium* accumulates certain metals proportionally to their concentration in soil and thus might provide information on the concentration of these elements present in the environment.

## Material and methods

### Sampling design

**Fig. 1:**
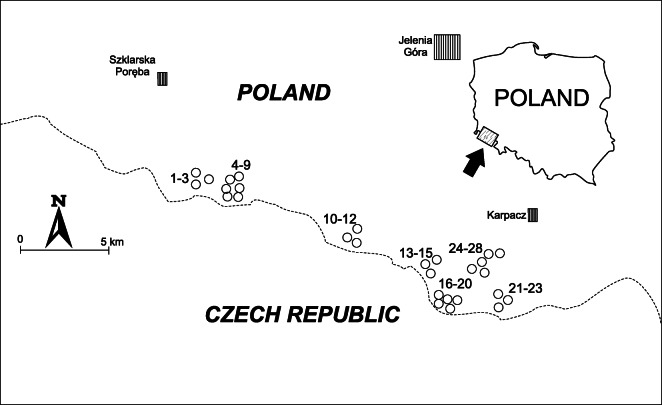
Location of the sampling sites

The investigation was carried out in the Karkonosze National Park (Fig. 1). Vital fronds of *A. distentifolium* as well as non-vital fronds from 28 sites were collected in early July (referred to as summer). Fronds turning brown were collected in early October (referred to as autumn). Collecting rhizomes in the Karkonosze National Park was not allowed, and the comparison of metal concentrations in summer and autumn fronds was expected to demonstrate which metals were withdrawn to the decaying fronds to protect the fern from their toxic influence. Samples were collected from open space not influenced by throughfall from canopies (Markert et al. 1996; Harmens et al. 2004) in mountain pasture/meadow below Łabski Peak (sites 1–3), Mały Śnieżny Cirque (sites 4–6), Duży Śnieżny Cirque (sites 7–9), Odrodzenie mountain hotel (sites 10–12), Wielki Staw Cirque (sites 13–15), Mały Staw Cirque (sites 16–20), Łomniczka Cirque (sites 21–23), and Domek Myśliwski Karkonosze National Park Ecological Education Centre and in the vicinity of walking trails (sites 24–28). The geographical coordinates of the sample locations are given in ESM 1. The samples were divided into two groups: (1) collected on the border of the upper forest belt and subalpine belt (sites 10–12, 24–28) and (2) collected in the subalpine belt (7–9, 13–15, 16–20, 21–23). At each site, three replicates of fronds, both vital and non-vital (where present), were collected at random within a 25 m × 25 m square. Additionally, samples of topsoil (0–10-cm depth) were collected (3 replicates) from each square in summer. The metal level in unpolluted soils depends on factors, such as parent rock geochemistry (Kabata-Pendias 2001a). However, the Karkonosze soils were influenced by long-range fallout contamination (Kabała and Szerszeń 2002). Thus, the evaluation of metal reference levels was difficult. In all the examined sites, parent rock consisted of granite, and it may be supposed that the geochemistry of soil developed on this granite was comparable in the investigated area (Waroszewski et al. 2013). Soil pH was acidic (Table 1). Fronds were not washed but cleaned of litter and mineral particles (Aboal et al. 2004; Oliva and Rautio 2004).

**Table 1:** Minimum and maximum values, median and average deviation (AD) of summer concentrations (mg kg^−1^) of total and available elements for plants in soil from the Karkonosze

Element	Minimum	Maximum	Median	AD		Minimum	Maximum	Median	AD
	Total				Threshold	Available			
pH	3.8	4.9	4.3	0.2					
Cd	0.1	1.0	0.3	0.2		0.01	0.5	0.1	0.07
Co	0.04	3.6	0.8	0.7	≤ 5	0.01	0.5	0.1	0.09
Cr	0.2	14	5.6	2.4	≤ 100	0.04	0.7	0.2	0.13
Cu	6.8	68	33	13	≤ 100	0.81	27	7.2	3.69
Fe	833	14,440	5530	2530		168	2689	701	305
Mn	9.5	280	92	52	≤ 1500	1.1	86	15	14
Ni	0.9	9.9	3.7	13	≤ 100	0.02	3.2	0.5	0.58
Pb	13	252	99	47	≤ 100	18	177	51	30
Zn	8.4	105	44	17	≤ 300	0.9	58	4.3	8.7

The data in the column threshold are environmental limits admissible for clean soil (Kabata-Pendias 2001)

### Soil and plant analysis

Soil particulates > 2 mm were removed with a Morek Multiserw LPzE-2e sieve shaker, and for homogenisation, a Fritsch Pulverisette mortar grinder was used. Dried frond samples were homogenised in a laboratory mill (IKA M20 Labortechnik). Both soil and frond samples were dried (50 °C) until constant weight. Plant available metals (Cd, Co, Cr, Cu, Fe, Mn, Ni, Pb and Zn) in soil were evaluated by extraction with 1 M ammonium acetate-EDTA (pH 4.65) for 30 min (10 g dry soil in 100 mL) (Cottenie et al. 1982). Total concentrations of metals in soil and concentration of metals in fronds were evaluated as follows: 300 mg of soil or plant samples (dry weight in triplicate) was digested with 65% ultrapure nitric acid (3 mL) and 70% ultrapure perchloric acid (2 mL) in a microwave oven (CEM Mars 5). Subsequently, deionised water was used for diluting the samples. The content of Fe, Mn and Zn was analysed by FAAS (Avanta PM from GBC) and Cd, Co, Cr, Cu, Ni and Pb by GFAAS (PinAAcle 900Z from Perkin-Elmer). All trace elements were controlled with Atomic Absorption Standard Solutions (Sigma Chemical Co.) and blanks containing the same matrix as the samples and were treated in the same way as the samples. Resulting metal contents were expressed on a dry weight basis. The accuracy of the methods was controlled with Chestnut Soil, Bainaimao and Bayan Obo, Neil Mongol in China GBW07402 (GSS-2) and Poaceae (mixture) IPE 952WEPAL Certified Reference Materials.

### Statistical analysis

One-way ANOVA (*p* < 0.05) followed by a post hoc Tukey test was applied to control the statistical significance of differences between sites with respect to the concentration of metals in soils and both types of fronds. Normal distribution was obtained by BoxCox transformation of data (Zar 1999). Shapiro-Wilk’s *W* test was applied for normality and the Brown-Forsythe test for the homogeneity of variances (Brown and Forsythe 1974; Argaç 2004). The difference in the content of metals between vital summer and autumn fronds was compared with the *t* test (Zar 1999). ANCOVA analysis was applied on BoxCox transformed data for comparison of the metal content in fronds of *A. distentifolium* in relation to the available metal content in soil, altitude of the sampling sites (group 1 and 2) and health status (vital vs non-vital).

To compare the accumulation of the same elements from soil by vital and non-vital *A. distentifolium*, an equal slopes model on BoxCox transformed data was applied. In this model, the independent variable (covariate) was the concentration of an element in soil, the dependent variable was the concentration of the same element in fronds of *A. distentifolium*, and the categorical predictor variable was the health status. The interaction between the health status and concentration of an element in soil tests the hypothesis that the slopes of regression lines were equal between vital and non-vital fronds. If the slopes were not significantly different, we further tested whether the intercepts were equal. When the slopes were significantly different, a model of heterogeneity of slopes was fitted. Interaction between the health status and concentration of an element in soil tests the unequal slopes hypothesis. In this model, the independent variable (covariate) was the concentration of an element in soil, the dependent variable was the concentration of the same element in *A. distentifolium*, and the categorical predictor variable was the health status (Snedecor and Cochran 1989; Quinn and Keough 2002).

Principal component and classification analysis (PCCA) was used for the evaluation of the similarity of Cd, Co, Cr, Cu, Fe, Mn, Ni, Pb and Zn contents in frond samples (Legendre and Legendre 1998). Co and Ni were applied as supplementary variables because of the lowest correlation coefficients with the 1st and 2nd factors for these elements (Zuur et al. 2007).

The bioaccumulation factor, BF, was calculated as the ratio between metal content in vital and non-vital summer fronds to plant available content in soil (Kłos 2009). The Mann-Whitney *U* test was applied for comparison of BF between vital and non-vital fronds.

The Dell Inc. version 13 (2015) program was applied for statistical evaluation.

## Results and discussion

One-way ANOVA showed that there were statistically significant differences in the concentration of the investigated metals in soils as well as fronds (both in summer and autumn samples) among the sites. The total maximum content of trace elements in soil (Table 1) exceeded the average values for unpolluted soils (mg kg^−1^): Cd < 0.5, Cu < 15, Pb < 25 and Zn < 70 (Kabata-Pendias 2001b). Soil from *A. distentifolium* sites contained higher upper concentrations of metals than the soil of the same species from Sudety valleys (Łomniczka and Sowia), Cd 0.1–0.5, Co 0.4–3.2, Cu 2.6–21, Mn 8–234 and Zn 13–62, and lower upper concentrations of Cr 1.3–20 and Fe 1416–20,100 (Krawczyk et al. 2006). Especially distinctive were total Pb contents which in some sites were higher than the admissible 100 mg kg^−1^ for cultivated soils (Table 1). High concentration of this element in Karkonosze soil was also reported by Kabała and Szerszeń (2002) and Wojtuń et al. (2017). Upper plant available soil concentrations of trace elements from *A. distentifolium* sites (Table 1) were higher for Cu, Mn, Ni and Pb and lower for Cd, Cr, Fe and Zn than those established by Wojtuń et al. (2017) for soils in the Karkonosze (mg kg^−1^): Cd 0.001–0.7, Cr 0.002–4.4, Cu 0.7–4.3, Fe 97–6220, Mn 0.2–46, Ni 0.002–2.1, Pb 12–77 and Zn 0.6–88. The plant available form of Pb in soil in some sites was also higher than the admissible level of this element for cultivated soils (Table 1).

The summer fronds of *A. distentifolium* from the Karkonosze subalpine belt (Table 2) contained higher upper concentrations of Cd, Cu, Fe, Ni and Zn and fronds collected from lower altitudes contained higher upper concentrations of Cd and Fe than ferns *Dryopteris filix-mas* and *Pteridium aquilinum* collected by Kozanecka et al. (2002) from an unpolluted nature reserve in Poland (Cd 0.3–0.8, Cr 0.4–1.7, Cu 6.4–13, Fe 58–115, Mn 60–1062, Ni 2.2–11 and Zn 11–58 in mg kg^−1^).

**Table 2:** Minimum and maximum values, median and average deviation (AD) of the summer concentration (mg kg^−1^) of trace elements in *Athyrium distentifolium* from group 1 (sites situated on the border of the upper forest belt and subalpine belt, no 10–12, 24–28) and group 2 (sites situated in the subalpine belt, no 7–9, 13–15, 16–20, 21–23)

	*A. distentifolium* 1				*A. distentifolium* 2			
	Minimum	Maximum	Median	AD	Minimum	Maximum	Median	AD
Cd	0.2	1.2	0.7	0.2	0.2	3.0	0.8	0.4
Co	< 0.04	0.2	0.04	0.03	< 0.04	0.4	0.1	0.1
Cr	0.03	0.6	0.3	0.2	0.1	0.6	0.2	0.1
Cu	5.5	10	7.1	0.9	5.7	24	8.9	2.3
Fe	18	154	63	35	51	185	71	20
Mn	123	814	253	160	117	1025	343	108
Ni	0.7	6.7	1.8	0.8	0.9	21	5.9	3.7
Pb	0.2	1.5	0.6	0.3	0.4	4.8	1.5	0.9
Zn	23	46	32	4.2	20	90	31	11

ANCOVA analysis (Table 3) revealed that the concentration of Pb in fronds was influenced by the content of these elements available for plants in soil, by altitude and by the health status. Cu, Fe and Ni concentrations in fronds were affected by altitude and the health status; the concentration of Mn in fronds was influenced by the content of Mn available for plants in soil and the health status, and the concentration of Co and Zn in fronds was influenced by the health status. Thus, the health status of ferns was influenced by the contents of Co, Cu, Fe, Mn, Ni, Pb and Zn in fronds (Table 3). Because no investigations into the problem of the dieback of *A. distentifolium* in the Karkonosze were performed, the explanation must be speculative. Plants may use elements accumulated in their tissues as defence against pathogens (Fones et al. 2010; Cheruiyot et al. 2013). For instance, Mn application may provide such protection to some plant species (Eskandari et al. 2018). Iron is reported as necessary for microbial pathogens thus organisms evolved mechanisms of suppressing this metal as protection against invasion by pathogens (Becker and Skaar 2014). Zinc is used in defence either by lowering the amount to deprive the pathogen of the metal or by increasing the amount to cause lethal effects in pathogens (Weiss and Carver 2018; Fones et al. 2019). Morkunas et al. (2018) report that both the hormetic effect (effect of metals at low doses) and toxic doses of trace metals may enhance plant resistance against pathogens. However, why non-vital fronds contained higher levels of certain metals needs further investigation. Results of simple linear regression showing a positive relation between plant available Mn and Pb in soil and in *A. distentifolium* for both vital and non-vital plants may give some insight. An equal slopes model showed that there was no significant interaction between the health status (vital and non-vital ferns) and concentration of Mn available in soil (*F*_1.80_ = 0.47, *p* = 0.49), indicated by homogeneous slopes of regression lines (ESM 2). This means that the relationship between the concentration of Mn available in soil and concentration of Mn in ferns was similar in vital and non-vital ferns. Significant differences in intercept were found among vital and non-vital ferns (*F*_1.81_ = 9.29, *p* < 0.0001): the regression line for non-vital ferns was higher than the line for vital ferns. This indicates that non-vital ferns would have higher Mn concentration at any concentration of Mn available in soil. An equal slopes model showed that there was a significant interaction between the health status (vital and non-vital ferns) and concentration of Pb available in soil (*F*_1.80_ = 4.89, *p* < 0.05), indicating heterogeneous slopes (ESM 3), that is, slopes of the regression lines were not parallel. This indicates that the relationship between Pb concentration in ferns and that available in soil differs between vital and non-vital ferns. The fitted regression line of Pb concentration in ferns to the concentration of Pb available in soil had a slope higher in non-vital than vital ferns (2.04 and 0.61, respectively). This indicates that Pb concentration in non-vital ferns increased more steeply with the concentration of Pb available in soil than in vital ferns (regression slope higher for the former). Therefore, non-vital ferns accumulated more Pb with the increasing concentration of Pb available in soil than vital ferns. Thus, the effect of the health status on Pb concentration in fronds was different for different concentrations of Pb available in soil. Above-mentioned results may point that higher accumulation of Pb in non-vital *A. distentifolium* may be caused by an increasing demand for protection against potential pathogens. Especially that ferns growing at higher altitudes, containing more Pb (Table 3), were much more healthy with less than 5% of the population with symptoms of dieback (K. Torzewski personal communication). However, for a better understanding of these processes, more investigation is required. The evaluated model may be applied for describing the mechanism of dependence between Pb concentration in soil and Pb in ferns depending on their health status.

**Table 3:** Analysis of covariance (ANCOVA, BoxCox transformed) for the effects of the health status (vital vs non-vital) and altitude of the sampling sites and variation among the sites in the concentration of plant available metals (mg kg^−1^) in soil and in the concentration of metals (mg kg^−1^) in fronds of *Athyrium distentifolium* in summer

		Df	MS	F	p			Df	MS	F	*p*
Cd	Soil	1	0.017	0.06	NS	Mn	Soil	1	1.98	9.01	< 0.01
	Health	1	0.11	0.38	NS		Health	1	2.68	12.21	< 0.001
	Altitude	1	1.57	5.42	< 0.05		Altitude	1	0.71	3.22	NS
Co	Soil	1	0.08	0.63	NS	Ni	Soil	1	0.09	0.20	NS
	Health	1	0.61	4.89	< 0.05		Health	1	6.91	16.06	< 0.001
	Altitude	1	0.27	2.12	NS		Altitude	1	14.04	32.64	< 0.001
Cr	Soil	1	3.89	19.02	< 0.001	Pb	Soil	1	8.37	24.10	< 0.001
	Health	1	0.66	3.23	NS		Health	1	2.07	5.96	< 0.05
	Altitude	1	0.22	1.06	NS		Altitude	1	13.64	39.28	< 0.001
Cu	Soil	1	0.0002	2.33	NS	Zn	Soil	1	0.0001	3.0	NS
	Health	1	0.00005	0.71	< 0.05		Health	1	0.0003	7.7	< 0.01
	Altitude	1	0.0009	12.65	< 0.001		Altitude	1	0.000001	0.0	NS
Fe	Soil	1	7.77	2.84	NS						
	Health	1	35.41	12.94	< 0.001						
	Altitude	1	27.38	10.00	< 0.01						

*NS* not significant

ANCOVA analysis also revealed that the concentration of Cd, Cu, Fe, Ni and Pb in fronds of *A. distentifolium* was influenced by the altitude of the sampling sites. The content of Cd, Ni and Zn in this species collected in the Tatra National Park was the highest in ferns of the same species growing at the highest altitudes (Samecka-Cymerman et al. 2012). An increased content of trace elements with altitude was also observed for 16 moss species investigated in the western Caucasus Mountains as well as in the Alps for *Pleurozium schreberi* and *Hylocomium splendens* (Zechmeister 1995; Shetekauri et al. 2015). In the mountains, the amounts of atmospheric metal deposition may vary locally. The bulk of dry and wet deposition in the sampling locations depends on their height above sea level (Gerdol and Bragazza 2006). This phenomenon in the high parts of the Karkonosze may be explained by anemo-orographic systems (Jeník 1961) favouring deposition of trace elements transported over long distances with high speed winds, among others (Kovář 2015).

Because it was difficult to distinguish vital and non-vital fronds in the autumn fronds, comparison with the *t* test was performed on trace element concentrations in summer (Table 4) and autumn plants (ESM 4) pooled together with no regard of the health status. The results show that the latter contained a significantly lower content of Zn (*p* < 0.001) and a significantly higher concentration of Cr, Fe, Mn, Pb (all at *p* < 0.001) and Cu (*p* < 0.05). The autumn fronds contained up to 3.2 times less Zn and up to 24, up to 3, up to 8, up to 7 and up to 65 times more Cr, Cu, Fe, Mn and Pb, respectively, than the fronds collected in summer. Thus, these metals seem to be sequestrated in senescing fronds probably to reduce the concentration of potentially harmful metals (Kachenko et al. 2007). Copper and Mn were not resorbed from senescing tissues to rhizomes by the fern *Dennstaedtia punctilobula* (Killingbeck et al. 2002). Iron and Mn are usually retained in older leaves of crop plants (Page and Feller 2015). The highest metal increase in autumn fronds of *A. distentifolium* from this investigation was observed for Pb. However, the concentration of this element in fronds was proportional to the concentration of Pb in soil. A similar correlation for Pb was found for *Athyrium wardii* by Zou et al. (2012). *Athyrium filix-femina* examined in beech forests in NW Germany accumulated Cd, Pb and Cu mostly in the rhizoids (Neite et al. 1991). Another member of Athyrium, *Athyrium yokoscense* (a Pb hyperaccumulator), also concentrated Pb in rhizoids. Similarly *Athyrium wardii* accumulated Pb in rhizoids with restricted translocation to fronds (Zou et al. 2012; Zhan et al. 2018). However, whether *A. distentifolium* has special tolerance for Pb like other members of the *Athyrium* genus needs further investigation.

**Table 4:** Minimum, maximum, median and average deviation (AD) of the summer concentration (mg kg^−1^) of trace elements in *Athyrium distentifolium* from vital and non-vital plants

	Vital				Non-vital			
	Minimum	Maximum	Median	AD	Minimum	Maximum	Median	AD
Cd	0.2	2.6	0.7	0.4	0.2	3.0	0.7	0.4
Co	< 0.04	0.3	0.01	0.04	< 0.04	0.4	0.1	0.1
Cr	0.03	0.6	0.2	0.1	0.1	0.6	0.2	0.1
Cu	5.7	15	8.0	1.4	5.5	24	8.4	3.2
Fe	18	181	67	19	54	185	73	26
Mn	117	585	317	101	185	1025	362	163
Ni	0.7	21	6.6	4.0	0.9	9.2	2.0	1.5
Pb	0.4	3.9	1.3	0.5	0.2	4.8	1.4	1.2
Zn	20	53	31	6.0	21	90	35	12

The ranges of the bioaccumulation factor BF (ratio of metal concentration in summer fronds to the concentration of available metal in soil) are presented in Table 5. The highest median value for both vital and non-vital ferns was calculated for Mn and the lowest for Pb. The Mann-Whitney *U* test revealed that vital ferns had a significantly higher BF for Ni (*p* < 0.05) and significantly lower for Pb (*p* < 0.01). There was no difference between vital and non-vital plants in BF for all other trace elements. Thus, it may be supposed that the disruption of tolerance mechanisms leading to elevated transport of the trace elements in non-vital fronds was not observed (Lange et al. 2016).

**Table 5:** Minimum, maximum, median of the bioaccumulation factor of trace elements in summer *Athyrium distentifolium* from vital and non-vital plants

	Vital			Non-vital		
	Minimum	Maximum	Median	Minimum	Maximum	Median
Cd	1.7	72	10	0.4	70	6.3
Co	0.001	2.9	0.5	0.001	7.0	0.7
Cr	0.08	12	0.6	0.2	5.3	1.1
Cu	0.3	9.5	1.1	0.6	7.5	1.4
Fe	0.02	0.4	0.1	0.02	0.2	0.1
Mn	3.8	217	21	5.5	420	25
Ni	0.43	221	12	0.5	21	4.5
Pb	0.005	0.1	0.02	0.01	0.1	0.03
Zn	0.5	39	8.1	0.4	44	7.5

The Tukey test distinguished homogenous groups of sites 7 and 21 with the lowest concentrations of trace metals in fronds and 1 and 16 with the highest concentrations of these elements. Sites 1 and 16 were situated in the vicinity of waste piles near hiking trails. Additionally, the heating system of the mountain hotel in Kocioł Małego Stawu affected site 16. Thus, the local sources of pollution within the Karkonosze National Park influenced metal contents in *A. distentifolium* fronds*.* The anthropogenic impact on *Polytrichum commune* and *Polytrichastrum formosum* from Karkonosze hotels was also reported by Wojtuń et al. (2018).

**Fig. 2:**
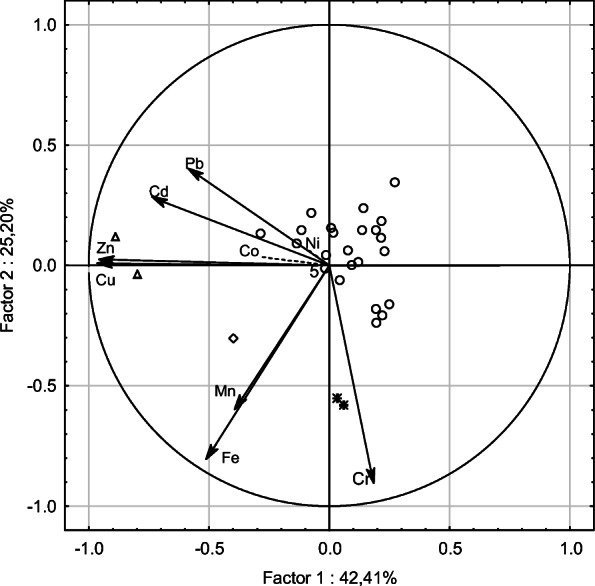
Ordination plot based on the concentrations of Cd, Cr, Cu, Fe, Mn, Pb and Zn (Co and Ni as supplementary variables) in *Athyrium distentifolium* fronds and projection of metal concentrations on the component plane. Triangles: sites 1 and 16. Diamond: sampling site 9. Stars: sites 24 and 25

Corresponding results may be observed in PCCA ordination established for metal contents in the summer fronds of *A. distentifolium* (Fig. 2). The first principal component discriminates sites 1 and 16 (negative scores). The second component discriminates sites 24 and 25 as well as site 9 (negative scores). Projection of the variables on the factor plane (Fig. 2) indicates that *A. distentifolium* from sites 1 and 16 was correlated with the highest concentrations of Cu and Zn in its tissues. *A. distentifolium* from sites 24 and 25 was correlated with the highest concentrations of Cr and Fe in its tissues. Site 9 was correlated with the highest concentrations of Cr, Cu, Fe and Zn. Sites 24 and 25 probably received metal contamination from the Karkonosze National Park Ecological Education Centre which uses an oil heating system. Site 9 was situated in Duży Śnieżny Kocioł influenced by the Karkonosze TV and radio relay building.

The presented investigation contributes to understanding the role of trace element pollution and behaviour in *A. distentifolium* growing in selected glacial cirques and their vicinity in the Karkonosze National Park and similar types of environment in the mountainous areas of Central Europe.

## Conclusions

1. *A. distentifolium* may use accumulated tissue elements as defence against pathogens.
2. *A. distentifolium* at subalpine elevations contained higher concentrations of Cd, Cu, Ni, Fe and Pb in fronds compared to lower elevations of upper forest belt.
3. *A. distentifolium* may sequestrate Cr, Cu, Fe, Mn and Pb in senescing fronds probably to remove potentially harmful metals.
4. Lead was the metal with the highest increase in autumn compared to summer fronds.
5. Non-vital and vital ferns accumulated Mn proportionally to the concentration in soil.
6. Non-vital ferns were better accumulators of Pb than vital ones which could be caused by an increasing demand for protection against potential pathogens.

## Electronic supplementary material

ESM 1 (PDF 134 kb)

ESM 2 (PDF 133 kb)

ESM 3 (PDF 72 kb)

ESM 4 (PDF 86 kb)
