# Supplementary material for: Trace elements in Athyrium distentifolium from alpine vegetation in the Karkonosze, SW Poland
Source: Environ Monit Assess. 2020 Jul 3;192(8):485. doi: 10.1007/s10661-020-08438-4 (PMC7332485; doi:10.1007/s10661-020-08438-4)

ESM 2. Relationship between Mn concentration in vital (circle, continuous line) and non-vital (crosses, dotted line) fronds of *Athyrium distentifolium* and concentration of Mn available in soil (BoxCox transformed).

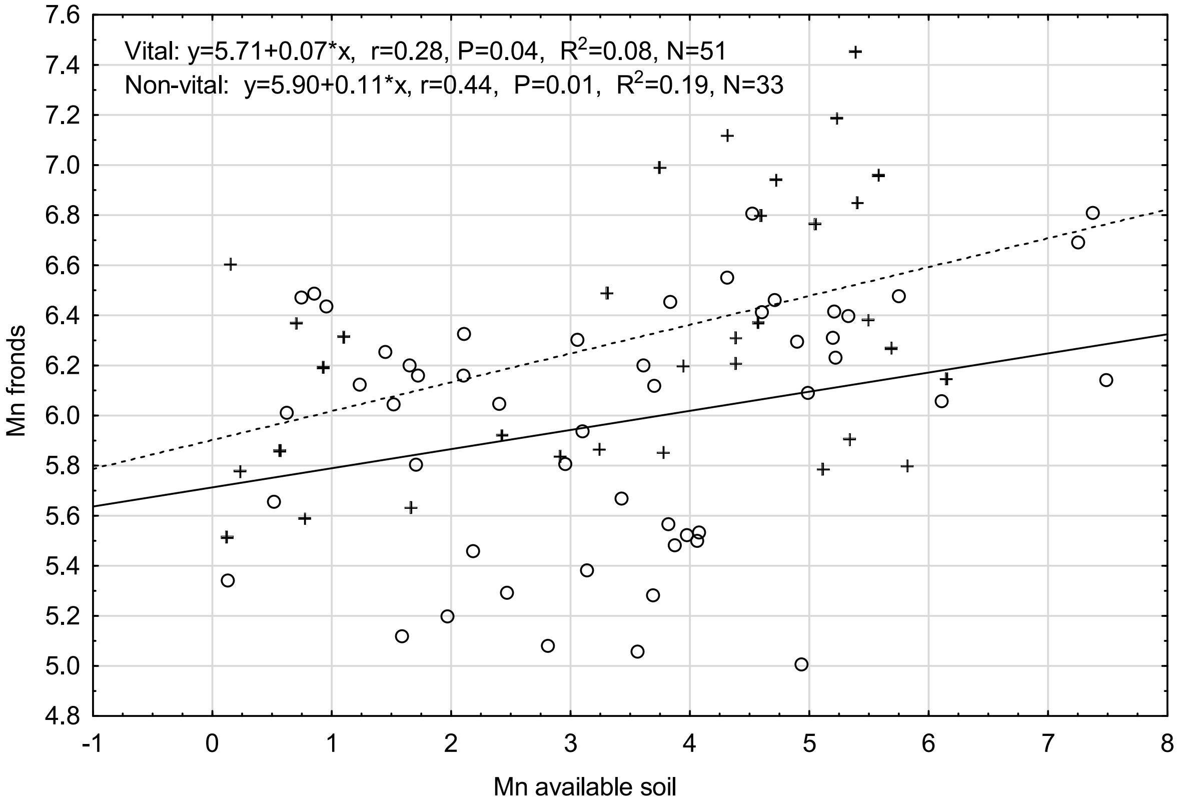

Supplement: Supplementary file 1 — (PDF 134 kb) [file 10661_2020_8438_MOESM1_ESM.pdf]
