# Supplementary material for: Trace elements in Athyrium distentifolium from alpine vegetation in the Karkonosze, SW Poland
Source: Environ Monit Assess. 2020 Jul 3;192(8):485. doi: 10.1007/s10661-020-08438-4 (PMC7332485; doi:10.1007/s10661-020-08438-4)
Supplement: Supplementary file 3 — (PDF 72 kb) [file 10661_2020_8438_MOESM3_ESM.pdf]

ESM 4. Minimum (Min), maximum (Max), median (Med) and average deviation (AD) of the autumn concentration ($mg \cdot kg^{-1}$) of trace elements in *Athyrium distentifolium* from the Karkonosze

	Minimum	Maximum	Median	AD
Cd	0.1	2.2	0.6	0.2
Co	<0.04	0.4	0.1	0.05
Cr	0.2	1.1	0.4	0.2
Cu	6.8	20	9.4	1.9
Fe	67	229	121	30
Mn	121	1983	429	224
Ni	0.4	22	3.5	3.8
Pb	2.6	66	16	12
Zn	8.6	55	23	9.3
