# Supplementary material for: Trace elements in Athyrium distentifolium from alpine vegetation in the Karkonosze, SW Poland
Source: Environ Monit Assess. 2020 Jul 3;192(8):485. doi: 10.1007/s10661-020-08438-4 (PMC7332485; doi:10.1007/s10661-020-08438-4)
Supplement: Supplementary file 4 — (PDF 86 kb) [file 10661_2020_8438_MOESM4_ESM.pdf]

ESM 1. Geographical coordinates of *Athyrium distentifolium* sampling sites

Sampling site	N	E	a.s.l.
	Łabski Cirque		
1	50°47’04.5’’	15°32’15.3	1270
2	50°47’06.7’’	15°32’23.7’’	1240
3	50°47’06.5’’	15°32’26.8’’	1250
	Mały Śnieżny Cirque		
4	50°46’56.0’’	15°33’25.7’’	1290
5	50°46’56.2’’	15°33’20.5’’	1350
6	50°46’51.9’’	15°33’15.9’’	1440
	Duży Śnieżny Cirque		
7	50°46’49.5’’	15°33’49.1’’	1270
8	50°46’44.7’’	15°33’45.6’’	1330
9	50°46’38.6’’	15°33’33.5’’	1460
	Odrodzenie mountain hotel		
10	50°45’55.0’’	15°38’07.4’’	1180
11	50°45’55.9’’	15°38’08.6’’	1170
12	50°45’56.9’’	15°38’09.3’’	1160
	Wielki Staw Cirque		
13	50°45’31.4’’	15°41’21.9’’	1270
14	50°45’27.9’’	15°41’24.4’’	1300
15	50°45’28.3’’	15°41’27.6’’	1270
	Mały Staw Cirque		
16	50°44’54.1’’	15°41’57.3’’	1185
17	50°44’55.6’’	15°41’54.5’’	1216
18	50°44’56.3’’	15°41’51.1’’	1263
19	50°44’54.3’’	15°41’50.6’’	1272
20	50°44’53.7’’	15°41’50.7’’	1274
	Łomniczka Cirque		
21	50°44’38.9’’	15°44’03.3’’	1130
22	50°44’30.4’’	15°43’55.2’’	1210
23	50°44’24.0’’	15°44’00.0’’	1260
	Domek Myśliwski and vicinity		
24	50°45’19.7	15°42’14.4’’	1140
25	50°45’21.0’’	15°42’16.3’’	1135
26	50°45’21.2’’	15°42’18.5’’	1140
27	50°45’41.6’’	15°42’15.4’’	1090
28	50°45’52.8’’	15°42’16.8’’	1075
